# Breast Milk Proteome: Changes in the Different Stages of Lactation and Impacts of Gestational Diabetes Mellitus and Body Mass Index

**DOI:** 10.1002/mnfr.70225

**Published:** 2025-09-01

**Authors:** Timo Seitz, Jenni Viitaharju, Chouaib Benchraka, Johannes Merilahti, Marko Kalliomäki, Lauri Polari, Diana Toivola, Leo Lahti, Otto Kauko, Kirsi Laitinen

**Affiliations:** ^1^ Nutrition and Food Research Center Faculty of Medicine University of Turku Turku Finland; ^2^ Food Sciences, Department of Life Technologies Faculty of Technology University of Turku Turku Finland; ^3^ Department of Obstetrics and Gynecology, Turku University Hospital Wellbeing Services County of Southwest Finland Turku Finland; ^4^ Institute of Biomedicine Integrative Physiology and Pharmacology Unit Faculty of Medicine University of Turku Turku Finland; ^5^ Department of Computing, Faculty of Technology University of Turku Turku Finland; ^6^ Turku Bioscience Centre University of Turku and Åbo Akademi University Turku Finland; ^7^ Department of Pediatrics, Faculty of Medicine Turku University Hospital Wellbeing Services County of Southwest Finland and University of Turku Turku Finland; ^8^ InFLAMES Research Flagship Center Turku Finland; ^9^ Åbo Akademi University Turku Finland

**Keywords:** BMI, breast milk, gestational diabetes mellitus, proteome

## Abstract

Breast milk proteome comprises hundreds of bioactive proteins supporting infant development. The extent to which maternal metabolic conditions modify the proteome is poorly known. This study investigates proteome evolution from colostrum to mature milk and examines the impacts of maternal gestational diabetes mellitus (GDM) and BMI on the proteome. We analyzed the proteome by data‐independent acquisition (DIA)‐based LC‐MS/MS from colostrum and mature milk samples collected from mothers (*n* = 47) with varying BMI values and with (*n* = 11) or without (*n* = 36) GDM. We identified 3496 proteins, of which 1055 were differentially abundant between colostrum and mature milk. Colostrum was enriched in proteins related to MHC II antigen response, intestinal IgA, adhesion, and glycosylation, while mature milk showed enrichment in lipid biosynthesis, protein translation, and degradation. In mature milk, mothers with both GDM and higher BMI had increased levels of proteins related to HDL formation. Proteins supporting the maturation of the naive gut immune system were prevalent in colostrum, while those prevalent in mature milk reflected the mammary gland's effort in biosynthesis. Both BMI and GDM have measurable effects on the breast milk proteome. The clinical significance of these findings on a child's future health needs to be clarified.

## Graphical abstract

We analyzed colostrum and breast milk from 47 mothers to see how proteins change over time and with maternal health. Colostrum had more immune proteins, while mature milk had proteins linked to growth support. Gestational diabetes and higher BMI altered the protein composition.

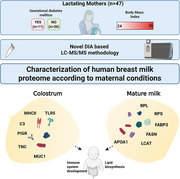

**Abbreviations**
ANOVA: analysis of variance
DA: differential abundance
DIA: data independent acquisition
FAIMS: high‐field asymmetric waveform ion mobility spectrometry
FC: fold change
FDR: false‐discovery‐rate
GDM: gestational diabetes mellitus
GO: gene Ontology
GSEA: gene set enrichment analysis
HF: high field
KEGG: Kyoto Encyclopedia of Genes and Genomes
O‐PLS‐DA: orthogonal partial least square discriminant analysis
OPLSR: orthogonal partial least square regression
PC: principal component
PCA: principal component analysis
TSE: TreeSummarizedExperiment
VIP: variable importance in projection

## 1 Introduction

Breast milk is the natural mode of feeding a newborn; it is important for the growth, development, and health of the child, with effects still evident when he/she reaches adulthood [1, 2]. Breast milk proteins are of particular interest as many are bioactive compounds; for example, immunoglobulins contribute to the development of the infant's immune system and protection from pathogens [3]. The breast milk protein content and composition vary with the stage of lactation, that is, from the first milk colostrum (1–5 days’ postpartum) to mature milk; this is due to the need to adapt to the evolving nutritional requirements of the infant [4, 5]. Nevertheless, the proteins in the breast milk, their longitudinal changes throughout the period of lactation, and further maternal factors, which could modify the composition, have not been fully studied with sophisticated proteomic techniques.

Previous studies utilizing comprehensive proteome analytics have identified from 739 [6] to as many as 2085 [7] proteins in breast milk with compositional changes taking place already during the first 2 weeks of lactation [8, 9]. The changes from the proteomes of colostrum to mature milk include some of the most abundant enzymes and/or transport and immunity proteins, for example, lactoferrin and immunoglobulins are the most abundant proteins present in colostrum [7, 10]. Using new advanced methods and high field asymmetric waveform ion mobility spectrometry (FAIMS) coupled with the Orbitrap Fusion Lumos, we recently identified a larger number, 2400 proteins, allowing a more detailed investigation of their potential biological and health impacts [11].

Gestational diabetes mellitus (GDM) and obesity are common conditions affecting the health of pregnant women and their children. In a previous study, the colostrum proteome was shown to be influenced by GDM as demonstrated by a downregulation of seven proteins and an upregulation of three proteins that are known to have an effect on the human immune system [12]. In a more recent study, 192 proteins were shown to differ between women with and without GDM [5]. Evidence for the effect of GDM on breast milk proteome is thus far limited and to our knowledge, there are no studies that have investigated the proteome of mature human milk.

In addition to the GDM, we anticipated that the presence of overweight or obesity in the mother could influence the breast milk proteome as elevated concentrations of inflammatory markers have been measured in 1‐ and 3‐month postpartum breast milk samples collected from women with obesity [13]. In another study, the levels of transforming growth factor β2 and soluble CD14, analyzed by enzyme‐linked immunosorbent assays, were reported to be lower in the breast milk of mothers with overweight as compared to mothers with normal weight [14]. In addition, the lower level of interferon γ in colostrum was associated with a higher body fat mass measured in late pregnancy [15]. Furthermore, the expression of 15 breast milk proteins, including the fragment of the sixth extracellular domain of the polymeric immunoglobulin receptor (PIGR), differed between mothers with and without obesity [16]. These results suggest that the protein composition, particularly that of the immunological proteins in breast milk, may be modulated by the metabolic health of the mother.

We hypothesized that there would be changes in the breast milk proteome during lactation, and furthermore that these would be influenced by the GDM status of mothers with overweight or obesity. The aims were to investigate the following: (1) the differences in the breast milk proteome and its functions between colostrum and mature milk, (2) the influence of the mother's GDM status on the colostrum and mature milk proteome, and (3) the relationship between GDM and body mass index (BMI) in determining the composition of the colostrum and mature milk proteome. We used an optimized proteomic methodology that improves not only the accuracy but also the depth of the analysis of the breast milk proteome [11].

## 2 Experimental Section

### 2.1 Study Population and Design

The breast milk samples were collected from mothers participating in a mother‐infant dietary single‐center intervention trial (clinicaltrials.gov, NCT01922791) being executed by Turku University Hospital and the University of Turku. The Ethics Committee of the Hospital District of Southwest Finland (115/180/2012) approved the study protocol, and the study met the guidelines of the Declaration of Helsinki 2013. All participants provided written informed consent. The study design has previously been described in detail [17]. Briefly, the study participants were recruited between October 2013 and July 2017, the inclusion criteria being the presence of overweight (BMI ≥ 25–30 kg/m^2^) or obesity (BMI > 30 kg/m^2^), early pregnancy (< 18 gestational weeks), and the absence of metabolic diseases. Here we have examined the colostrum samples collected at delivery in the hospital and breast milk samples collected when the infant was 3 months old. All the samples were from the placebo group, that is, from women who did not receive the dietary intervention.

At the gestational visits (13.9 SD ± 2.1 gestational weeks and 35.2 SD ± 0.9 gestational weeks), the women's heights and weights were measured, and their pre‐pregnancy BMI values were calculated using height and self‐reported pre‐pregnancy weight obtained from the records held in maternal welfare clinics. In addition, information on health, smoking habits, and obstetric medical history was obtained from the participants. The details of the pregnancy were obtained from medical records. GDM diagnoses were based on a 2‐h 75‐g oral glucose tolerance test if one or more values were at or above the threshold level: 0 h ≥ 5.3, 1 h ≥ 10.0, and 2 h ≥ 8.6 mmol/L, according to the Finnish Current Care Guidelines [18, 19]. An oral glucose tolerance test was performed in all women between 24 and 28 gestational weeks and to high‐risk women (BMI ≥ 35 kg/m^2^, previous GDM, glucosuria, polycystic ovarian syndrome, or family risk of diabetes) also at 12–16 gestational weeks.

### 2.2 Sample Collection and Preparation

The breast milk samples were collected by manual expression into 15 mL light‐protected polypropylene tubes. The colostrum samples were collected immediately after lactation had commenced (3.3 ± 1.2 days postpartum) in the maternity hospital and the mature milk (3 months’ postpartum) samples were collected at home. The samples were cooled down to +6°C and transferred to the study clinic and aliquoted and stored at −70°C prior to further analysis.

The samples were thawed, mixed, and centrifuged four times, once at 2000 × *g* for 20 min and three times at 10 000 × *g* for 10 min to remove fat. Between each centrifugation, the clear defatted supernatant was collected and moved to a new tube prior to further centrifugation. After the final centrifugation, the clear supernatant was aliquoted and stored at −80°C.

### 2.3 Mass Spectrometry Analysis

Human milk samples were denatured in 8 M urea in 50 mM Tris–HCl, pH 8. The samples were reduced with 10 mM D,L‐dithiothreitol, alkylated with 40 mM iodoacetamide, and then were digested overnight with sequencing grade modified trypsin (Promega). After digestion, the peptide samples were desalted with a Sep‐Pak tC18 96‐well plate (Waters) and evaporated to dryness. Digested peptide samples were dissolved in 0.1% formic acid, and peptide concentrations were determined with a NanoDrop device. Equal amounts of samples were analyzed on a nanoflow HPLC system (Easy‐nLC1200, Thermo Fisher Scientific) coupled to the Orbitrap Fusion Lumos mass spectrometer (MS) (Thermo Fisher Scientific, Bremen, Germany) equipped with a nano‐electrospray ionization source and FAIMS Pro interface. Peptides were first loaded on a trapping column and subsequently separated in line on a 15 cm C18 column (75 µm × 15 cm, ReproSil‐Pur 3 µm 120 Å C18‐AQ, Dr. Maisch HPLC GmbH, Ammerbuch‐Entringen, Germany). The mobile phase consisted of water with 0.1% formic acid (Solvent A) or acetonitrile/water (80:20 (v/v)) with 0.1% formic acid (Solvent B). A 120 min gradient was used to elute peptides (62 min from 5% to 21% Solvent B and 48 min from 21% to 36 % Solvent B, followed by a wash stage with 100% Solvent B). MS data were acquired automatically by using Thermo Xcalibur 4.4 software (Thermo Fisher Scientific). In the data independent acquisition (DIA) method, FAIMS compensation voltages −50 and −70 V were used and a duty cycle contained one full scan (resolution 120 000, AGC target 7E5, maximum injection time 50 ms, 400–1000 m/z) and 30 DIA MS/MS scans (resolution 30 000, AGC target 1e6, maximum injection time 52 ms) covering the mass range 400–1000 with variable width isolation windows.

### 2.4 Protein Identification and Quantification Analysis

Data were analyzed by Spectronaut software (Biognosys; version 16.0.220606.53000); the analysis consisted of identifications of protein and label‐free quantifications of protein abundances. A direct DIA approach was used to identify proteins and label‐free quantifications were performed with MaxLFQ. The main data analysis parameters in Spectronaut were the following: (i) enzyme: Trypsin/P, (ii) missed cleavages: 2, (iii) fixed modifications: carbamidomethyl (cysteine), (iv) variable modifications: acetyl (protein N‐terminus) and oxidation (methionine), (v) protein database: Homo Sapiens Swiss‐Prot reference proteome (Uniprot release 2021_04), and (vi) normalization: local normalization.

### 2.5 Data Analysis

The primary outcomes of the main study regarding the impact of the dietary intervention on the risk of GDM and child allergy have been reported earlier [17, 20], and the results presented here are predefined secondary outcomes. Baseline characteristics between the mothers with or without GDM were compared using one‐way analysis of variance (ANOVA) or χ2 test and continuous variables were summarized as medians with interquartile ranges and categorical variables with counts (*n*) and percentages; the statistical analysis was performed using IBM SPSS Statistics for Windows, version 27 (Chicago, IL, USA) (DA). The construction of a TreeSummarizedExperiment (TSE) object [21], integrating protein abundances with clinical metadata for downstream analysis, was performed using the R package *mia* (v1.15), *miaViz* (v1.10), and *ggplot2* (v3.5.1) [22, 23, 24]. Principal component analysis (PCA) and PERMANOVA were performed on CLR‐transformed data and used to test for group separation of the lactational stages with respect to the proteome composition and the GDM status, as well as for BMI. PERMANOVA was conducted using the *vegan* package (v2.6.8), including the β‐dispersion test for homogeneity. Differentially abundant (DA) proteins were screened with the R packages *lme4* (v1.1.35.3) comparing colostrum and mature milk to account for the paired sample design and *limma* (v3.58.1) for the GDM status and BMI within colostrum and mature milk to obtain *p* values followed by multiple correction with the Benjamini–Hochberg method and filtered by log2 fold change (adj. *p* < 0.05, abs.log2FC ≥ 1) [25, 26]. For the differential abundance analysis using *lme4*, only proteins with a prevalence of ≥ 90% in at least colostrum or mature milk were included. The analysis accounted for the sample pairing (mothers were included as a stratum) with BMI being applied as a covariate. All proteins were included in the analysis using *limma*, maximizing discovery potential.

The Gene Ontology (GO) annotation, Kyoto Encyclopedia of Genes and Genomes (KEGG) pathway, Reactome database, and R package *ClusterProfiler* (v4.10.1) carried out the Gene Set Enrichment Analysis (GSEA) of differentially regulated proteins ranked by *p* value [27, 28, 29]. GSEA included all proteins, for single protein discussion ≥ 90% prevalent proteins were considered. Due to the large number of proteins measured, it should be noted that proteins that display log2FC do not often yield significant results in statistical tests of large‐scale omics experiments due to the large penalty for multiple correction [30]. Therefore, in the exploratory feature selection, the proteins were filtered (*p* ≤ 0.05, for GDM status abs. log2FC ≥ 1, and for BMI abs. log2FC ≥ 0.1).

## 3 Results

### 3.1 Study Population

The baseline clinical characteristics of the women are summarized in Table 1. The majority (72%) of the participants were overweight, and the remaining 28% were obese. Most were multipara (53%) and had had a university or college education (60%). No differences in the clinical and demographic parameters between women with and without GDM were observed.

**TABLE 1:** Clinical characteristics of all mothers with overweight/obesity and according to gestational diabetes mellitus (GDM) status. The results are presented as mean ± SD or *n* (%).

Characteristics	All, *n *= 47	GDM, *n* = 11	without GDM, *n* = 36	*p* value
Age	31.3 ± 3.5	31.5 ± 4.0	31.3 ± 3.4	0.876^a^
University or college education	31 (66)	5 (45)	26 (72)	0.101^b^
Prepregnancy BMI (kg/m^2^)	28.8 ± 2.8	27.6 ± 2.3	29.2 ± 2.8	0.098^a^
Primipara	22 (47)	4 (36)	18 (50)	0.428^b^
Allergy, asthma, atopy	13 (28)	3 (27)	10 (28)	0.852^b^
Family history of diabetes	9 (19)	3 (27)	6 (17)	0.642^b^

^a^ One‐way ANOVA.

^b^ χ2 test.

### 3.2 The Global Proteome of Breast Milk Differs Throughout Lactation, Whereas GDM and BMI Have Little Effect on the Proteome of Either Colostrum or Mature Milk

**FIGURE 1:**
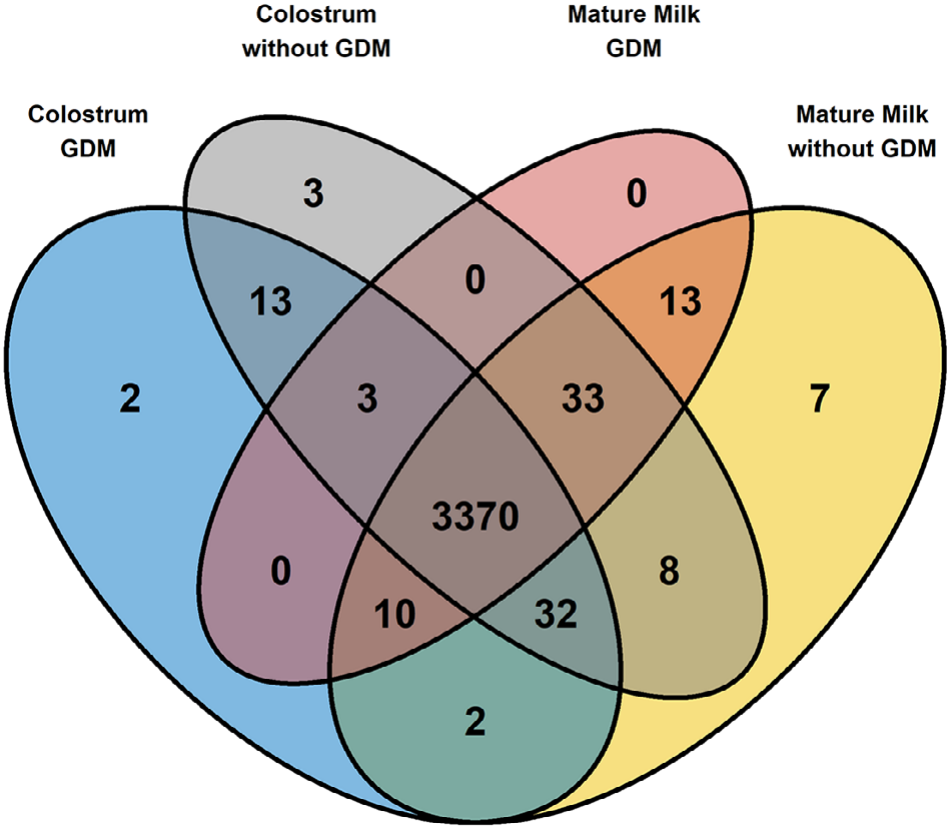
Venn diagram showing common proteins among colostrum and mature milk samples from women with or without GDM.

**FIGURE 2:**
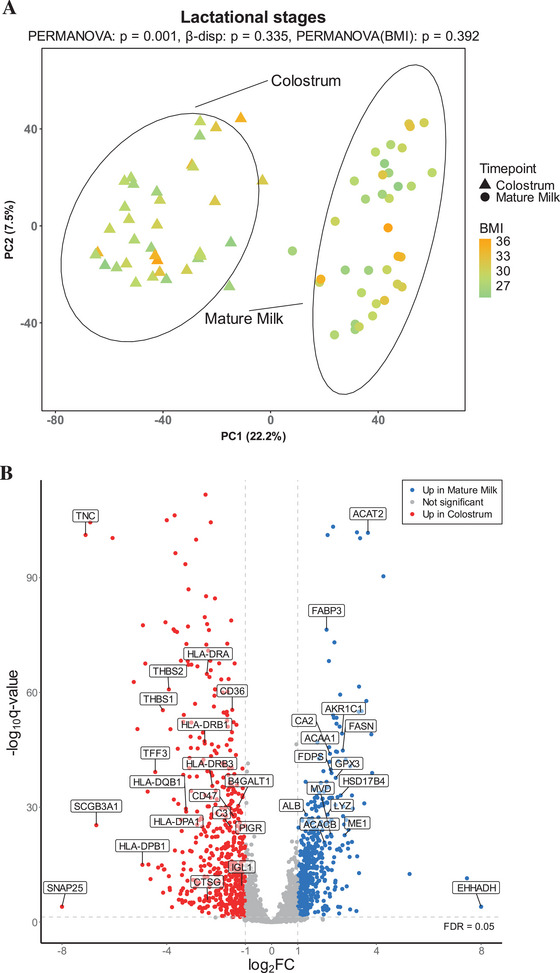
Comparison of colostrum and mature milk. (A) PCA displaying the proteomic landscape of the breast milk samples with respect to the lactational stage (triangles = colostrum; circles = mature milk) and the BMI status (orange‐green scale). (B) Volcano plot of differentially abundant proteins between colostrum and mature milk. Red color indicates proteins upregulated in colostrum (log2FC ≤ −1 and adj. *p* ≤ 0.05) and blue color indicates proteins upregulated in mature milk (log2FC ≥ 1 and adj. *p* ≤ 0.05).

In total, 3496 different proteins were identified in the breast milk samples (Figure 1). Among all colostrum samples, we identified 18 proteins not found in mature milk. Of those, two proteins achieved an identification rate exceeding 80%: pregnancy‐specific beta‐1‐glycoprotein 2, PSG2 (95.7%), and C‐X‐C motif chemokine ligand 13, CXCL13 (86%) (Figure 2A). From the 20 unique proteins detected in mature milk, the highest identification rate was obtained for keratin 79, KRT79 (55%), and the component of oligomeric Golgi complex 8, COG8 (30%) (Figure 2B).

**TABLE 2:** Top 10 proteins ranked by their expression in colostrum and mature milk and their regulation between colostrum and mature milk (lactational stage) and with respect to the participants’ GDM status within colostrum and mature milk. The *p* values retrieved from the limma analysis.

								GDM status					
Protein			Rank		Lactational stages			Colostrum			Mature milk		
Gene name	Uniprot	Description	Colostrum	Mature milk	log2FC	*p*	adj. *p*	log2FC	*p*	adj. *p*	log2FC	*p*	adj. *p*
LTF	P02788	Lactotransferrin	1	1	−0.22	0.006	0.029	0.20	0.24	0.58	−0.12	0.48	0.95
CSN3	P07498	Casein kappa	2	2	0.67	1E‐13	2E‐12	0.08	0.67	0.85	−0.08	0.66	0.97
LALBA	P00709	Lactalbumin alpha	3	3	0.61	8E‐11	8E‐10	0.13	0.39	0.68	−0.05	0.82	0.98
PIGR	P01833	Polymeric immunoglobulin receptor	4	7	−1.17	3E‐24	7E‐23	0.52	0.02	0.50	−0.06	0.77	0.97
ALB	P02768	Albumin	5	4	1.33	3E‐30	9E‐29	0.19	0.37	0.67	−0.12	0.47	0.95
IGHA1	P01876	Immunoglobulin heavy constant alpha 1	6	10	−0.74	3E‐06	2E‐05	0.42	0.30	0.64	−0.17	0.50	0.95
IGL1_HUMAN	P0DOX8	Immunoglobulin lambda‐1 light chain	7	12	−1.14	4E‐11	4E‐10	0.47	0.34	1.00	−0.07	0.77	0.97
CEL	P19835	Bile salt‐stimulated lipase (BSSL)	8	6	1.62	1E‐21	2E‐20	−0.05	0.89	0.95	−0.01	0.96	0.99
CSN2	P05814	Casein beta	9	8	0.54	2E‐05	0.0001	0.17	0.39	0.69	−0.03	0.92	0.99
TNC	P24821	Tenascin C	10	154	−7.16	1E‐104	7E‐102	0.08	0.82	0.92	−0.53	0.49	0.95
LYZ	P61626	Lysozyme	14	5	2.15	2E‐30	8E‐29	0.33	0.34	0.66	0.05	0.90	0.99
FABP3	P05413	Fatty acid binding protein 3	24	9	2.11	2E‐79	4E‐77	−0.08	0.68	0.85	0.48	0.04	0.94

In addition, 14 unique proteins were identified in colostrum samples from mothers with GDM and 44 in those without GDM, while in mature milk, 3 unique proteins were found in samples from mothers with GDM and 49 in those without this disorder (Figure 1). Unique proteins in colostrum or mature milk from mothers with or without GDM had an identification rate of less than 40% (Table S1).

With respect to the highly expressed proteins, lactotransferrin, κ‐casein, and α‐lactalbumin were the most abundant in both colostrum and mature milk, with no changes observed between colostrum and mature milk (adj. *p* ≤ 0.05 log2FC ≥ 1 or ≤ −1, Table 2). When the highly expressed proteins in colostrum and mature milk were compared, the largest statistically significant changes were evident in tenascin C (TNC, log2FC = −7.15), lysozyme (LYZ, log2FC = 2.15), and fatty acid binding protein 3 (FABP3, log2FC = 2.11). Despite the change between the timepoints, the expression of lysozyme and FABP3 remained high in both stages in comparison with all proteins, whereas the expression of TNC declined in the ranking of the highly expressed proteins from colostrum to mature milk (Table 2).

**FIGURE 3:**
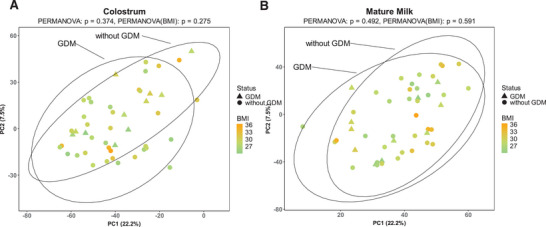
Comparison of mothers with and without GDM. PCA displaying the proteomic landscape in (A) colostrum and (B) mature milk samples (triangles = with GDM; circle = without GDM; orange‐green scale  =  BMI).

Principal component analysis (PCA) revealed the separation between the colostrum and mature milk proteome. This was confirmed with PERMANOVA (*p* = 0.001) and the β‐dispersion test (*p* = 0.3) (Figure 2). When colostrum and mature milk were inspected separately (shared PCA ordination but shown separately for the two groups), no distinct groupings based on the GDM status in either colostrum (*p* = 0.3) or mature milk (*p* = 0.5) were visible in PCA visualization (Figure 3). No statistically significant association with BMI was observed in the PCA ordination in either colostrum (*p* = 0.3) or mature milk (*p* = 0.6).

### 3.3 Changes in the Proteome From Colostrum to Mature Milk

In all, 1055 differently abundant proteins were observed in the comparison between colostrum and mature milk (adj. *p* < 0.05, log2FC ≥ 1 and ≤ −1); 571 proteins were upregulated in colostrum; and 484 were upregulated in mature milk (Figure 2B, Table S2).

**FIGURE 4:**
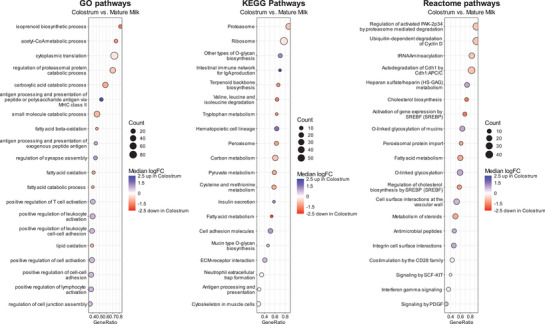
Gene Set Enrichment Analysis (GSEA) of differentially expressed proteins between colostrum and mature milk. The dot plots illustrate enriched pathways comparing the Gene Ontology (GO), Kyoto Encyclopedia of Genes and Genomes (KEGG), and Reactome database of the GSEA of colostrum versus mature milk. Each pathway is represented by a dot, where the size corresponds to the count of differentially expressed proteins, and the color scale indicates the median log fold change (logFC). Pathways ranked by GeneRatio and median logFC highlight the degree and directionality of regulation (blue: upregulated; red: downregulated). All pathways between colostrum and mature milk are significant (*p* < 0.05). GSEA results, including all pathways provided in the Supporting Information.

The subsequent GSEA of enriched up‐ and downregulated proteins revealed a total of 597 GO, KEGG, and Reactome pathways that showed statistically significant (adj. *p* < 0.05) changes from colostrum to mature milk (Table S3). All three databases highlighted the upregulation of the immune response and cell adhesion‐related processes such as antigen processing and presentation via MHC class II, intestinal immune network for IgA production, positive regulation of cell–cell adhesion, O‐glycan biosynthesis, O‐linked glycosylation of mucins, and integrin cell surface interaction in colostrum (Figure 4). The majority of the proteins in these pathways included members of the MHC class II complex, immunoglobulins, cell surface proteins classified as a cluster of differentiation (CD), glycosyltransferases, extracellular matrix proteins, and integrins (Table 3). In mature milk, the vast majority of the upregulated pathways indicated by all databases were categorized into the metabolism of lipids, translation, and protein degradation (Figure 4). The lipid‐associated pathways included those involved in fatty acid and cholesterol biosynthesis, which encompassed the more specific pathway of isoprenoid biosynthetic processes and terpenoid backbone biosynthesis, which is tied to the upregulation of the multienzyme protein fatty acid synthase (FASN), fatty acid binding protein 3 (FABP3), acyltransferases, dehydrogenases, synthases, and synthetases (Table 4). The translational processes included tRNA aminoacylation, ribosome, and cytoplasmic translation, which was driven by the proteins related to the small and large ribosomal subunit proteins (Table 4). Protein degradation was related to the upregulation of several pathways, for example, regulation of the proteasomal protein catabolic processes, proteasome, regulation of activated PAK2p34 by proteasome‐mediated degradation, and this included the proteins of the subunits of the 26S and 20S proteasome (Table 4). Neither the proteins with high levels in colostrum nor those high in mature milk exhibited statistically significant associations with the presence of GDM or with the BMI (Tables 3 and 4).

**TABLE 3:** Top 50 proteins upregulated in colostrum compared with mature milk ranked by the percentage of total pathways (%_TP_) in which each protein is involved from the GSEA. Additionally, the regulation of these proteins in colostrum of mothers with GDM in comparison to those without this disorder and by varying BMI values. Proteins shown are *p* adj. < 0.05, ≥ 5%_TP_, log2FC ≤ −1, top 50 by log2FC, and ranked by %TP from the comparison of colostrum with mature milk (full table in Supporting Information S7).

Protein				Higher in colostrum			GDM			BMI		
Gene name	Uniprot	Description	%_TP_	log2FC	*p*	adj. *p*	log2FC	*p*	adj. *p*	log2FC	*p*	adj. *p*
HLA‐DRB1	P01911	Major histocompatibility complex, class II, DR beta 1	38.9	−2.55	3E‐49	2E‐47	0.40	0.23	0.58	−0.40	0.22	0.78
HLA‐DRA	P01903	Major histocompatibility complex, class II, DR alpha	32.2	−2.47	1E‐67	1E‐65	0.27	0.29	0.63	−0.31	0.22	0.78
HLA‐DPA1	P20036	Major histocompatibility complex, class II, DP alpha 1	30.7	−3.46	6E‐26	2E‐24	0.34	0.39	0.69	−0.37	0.35	0.81
HLA‐DPB1	P04440	Major histocompatibility complex, class II, DP beta 1	30.4	−4.94	7E‐17	1E‐15	0.24	0.65	0.84	−0.45	0.40	0.82
HLA‐DRB3	P79483	Major histocompatibility complex, class II, DR beta 3	29.5	−2.27	6E‐38	3E‐36	0.25	0.45	0.72	−0.33	0.31	0.79
HLA‐DQB1	P01920	Major histocompatibility complex, class II, DQ beta 1	28.1	−3.28	4E‐31	1E‐29	−0.13	0.81	0.91	−0.92	0.08	0.73
SRC	P12931	SRC proto‐oncogene, non‐receptor tyrosine kinase	24.3	−2.46	1E‐16	2E‐15	0.39	0.32	0.66	−0.40	0.30	0.79
WNT5A	P41221	Wnt family member 5A	21.9	−3.07	8E‐55	7E‐53	−0.10	0.70	0.87	−0.34	0.16	0.75
CD74	P04233	CD74 molecule	21.1	−3.16	2E‐45	1E‐43	0.99	0.03	0.50	−0.88	0.05	0.73
THBS1	P07996	Thrombospondin 1	18.5	−4.15	4E‐58	4E‐56	0.24	0.49	0.75	−0.35	0.30	0.79
ELANE	P08246	Elastase, neutrophil expressed	16.4	−3.82	1E‐10	1E‐09	−0.38	0.66	0.84	0.77	0.36	0.81
COL4A1	P02462	Collagen type IV alpha 1 chain	16.2	−3.42	5E‐55	5E‐53	−0.51	0.21	0.57	−0.28	0.48	0.84
C3	P01024	Complement C3	15.9	−1.57	5E‐27	1E‐25	0.53	0.09	0.51	−0.61	0.05	0.73
COL4A2	P08572	Collagen type IV alpha 2 chain	15.1	−3.72	5E‐110	5E‐107	−0.46	0.23	0.58	−0.23	0.53	0.85
CTSG	P08311	Cathepsin G	15.0	−2.39	3E‐07	2E‐06	−0.20	0.79	0.91	0.97	0.21	0.78
ITGA6	P23229	Integrin subunit alpha 6	15.0	−1.58	5E‐26	1E‐24	0.35	0.14	0.52	−0.33	0.17	0.75
HSPG2	P98160	Heparan sulfate proteoglycan 2	14.3	−2.60	6E‐45	4E‐43	0.40	0.27	0.61	−0.50	0.16	0.75
SDC1	P18827	Syndecan 1	14.1	−1.57	3E‐10	3E‐09	0.40	0.45	0.72	−0.51	0.33	0.80
AGRN	O00468	Agrin	14.0	−1.74	5E‐19	9E‐18	0.64	0.12	0.51	−0.68	0.09	0.73
VCAM1	P19320	Vascular cell adhesion molecule 1	13.9	−1.88	7E‐20	1E‐18	0.40	0.45	0.73	−0.28	0.59	0.87
CD55	P08174	CD55 molecule (Cromer blood group)	13.8	−1.41	2E‐31	6E‐30	0.20	0.36	0.67	−0.18	0.39	0.82
CD36	P16671	CD36 molecule (CD36 blood group)	13.8	−1.49	3E‐58	4E‐56	0.36	0.05	0.50	−0.29	0.10	0.74
MMP9	P14780	Matrix metallopeptidase 9	13.6	−3.36	7E‐10	6E‐09	0.02	0.98	1.00	0.99	0.25	0.78
KIT	P10721	KIT proto‐oncogene, receptor tyrosine kinase	12.8	−1.66	1E‐12	1E‐11	0.55	0.08	0.50	−0.42	0.18	0.76
COL5A2	P05997	Collagen type V alpha 2 chain	12.4	−2.36	2E‐32	6E‐31	−0.63	0.14	0.52	−0.30	0.47	0.84
COL4A5	P29400	Collagen type IV alpha 5 chain	12.3	−3.25	7E‐32	3E‐30	−0.35	0.40	0.69	−0.27	0.50	0.84
CD47	Q08722	CD47 molecule	12.1	−1.43	8E‐30	3E‐28	0.31	0.16	0.53	−0.47	0.04	0.73
LAMA1	P25391	Laminin subunit alpha 1	11.3	−1.69	1E‐19	2E‐18	0.07	0.83	0.92	0.09	0.79	0.94
TNC	P24821	Tenascin C	10.2	−7.16	1E‐104	7E‐102	0.08	0.82	0.92	−0.19	0.57	0.87
GNAI2	P04899	G protein subunit alpha i2	9.9	−2.47	2E‐88	7E‐86	0.11	0.55	0.78	−0.01	0.95	0.99
ST3GAL1	Q11201	ST3 beta‐galactoside alpha‐2,3‐sialyltransferase 1	9.9	−1.33	3E‐27	8E‐26	0.17	0.48	0.74	−0.22	0.35	0.81
THBS2	P35442	Thrombospondin 2	9.7	−3.93	1E‐63	2E‐61	0.18	0.60	0.81	−0.08	0.82	0.95
FBN1	P35555	Fibrillin 1	9.2	−3.54	4E‐33	1E‐31	0.58	0.36	0.67	−0.71	0.26	0.78
FZD7	O75084	Frizzled class receptor 7	9.2	−2.33	2E‐29	5E‐28	0.28	0.47	0.74	−0.20	0.60	0.87
CAMP	P49913	Cathelicidin antimicrobial peptide	9.1	−2.71	1E‐07	8E‐07	0.11	0.89	0.95	1.12	0.16	0.75
ST6GAL1	P15907	ST6 beta‐galactoside alpha‐2,6‐sialyltransferase 1	9.0	−1.47	6E−24	1E−22	−0.29	0.37	0.67	0.22	0.48	0.84
COL6A1	P12109	Collagen type VI alpha 1 chain	8.8	−1.49	1E−13	2E−12	0.17	0.74	0.89	−0.21	0.68	0.90
ADCY3	O60266	Adenylate cyclase 3	8.6	−1.52	5E‐34	2E‐32	0.21	0.39	0.69	−0.12	0.63	0.88
GNAI1	P63096	G protein subunit alpha i1	8.1	−3.00	9E‐13	1E‐11	0.37	0.23	0.58	−0.45	0.14	0.75
GNA11	P29992	G protein subunit alpha 11	7.2	−1.44	6E‐55	6E‐53	0.11	0.56	0.79	−0.16	0.42	0.82
B4GALT1	P15291	Beta‐1,4‐galactosyltransferase 1	7.2	−1.25	1E‐32	4E‐31	0.48	0.05	0.50	−0.44	0.06	0.73
FZD1	Q9UP38	Frizzled class receptor 1	7.1	−3.18	3E‐90	1E‐87	0.38	0.31	0.65	−0.51	0.17	0.76
GALNT1	Q10472	Polypeptide N‐acetylgalactosaminyltransferase 1	7.0	−2.00	1E‐70	2E‐68	0.55	0.03	0.50	−0.39	0.11	0.74
VIM	P08670	Vimentin	6.7	−2.80	2E‐11	2E‐10	−0.21	0.77	0.90	1.04	0.15	0.75
PLOD3	O60568	Procollagen‐lysine,2‐oxoglutarate 5‐dioxygenase 3	6.3	−2.63	7E‐130	1E‐126	0.12	0.58	0.80	−0.29	0.18	0.76
H2BC11	P06899	H2B clustered histone 11	6.3	−2.75	3E‐07	2E‐06	−0.25	0.69	0.86	0.88	0.16	0.75
DEFA3	P59666	Defensin alpha 3	6.0	−4.19	1E‐16	2E‐15	−0.30	0.72	0.88	1.18	0.16	0.75
H4C4	P62805	H4 clustered histone 4	5.8	−1.89	7E‐07	5E‐06	−0.39	0.51	0.76	1.43	0.02	0.73
SNAP25	P60880	Synaptosome associated protein 25	5.7	−8.00	1E‐04	0.0001	0.89	0.08	0.50	−0.77	0.15	0.75
GALNT16	Q8N428	Polypeptide N‐acetylgalactosaminyltransferase 16	5.4	−3.81	4E‐53	4E‐51	0.63	0.07	0.50	−0.77	0.02	0.73

**TABLE 4:** Top 50 proteins upregulated in mature milk compared with colostrum ranked by the percentage of total pathways (%_TP_) in which each protein is involved from the GSEA. Additionally, regulation of these proteins in mature milk of mothers with GDM compared to those without this disorder, and by varying BMI values. Proteins shown are *p* adj. < 0.05, ≥ 5%_TP_, log2FC ≥ 1, top 50 by log2FC, and ranked by %_TP_ from the comparison of colostrum with mature milk (full table in Supporting Information S7).

Protein			%_TP_	Higher in mature milk			GDM			BMI		
Gene name	Uniprot	Description		log2FC	*p*	adj. *p*	log2FC	*p*	adj. *p*	log2FC	*p*	adj. *p*
ACAT2	Q9BWD1	Acetyl‐CoA acetyltransferase 2	25.5	3.67	3E‐105	2E‐102	−0.46	0.11	0.51	−0.16	0.58	0.99
EHHADH	Q08426	Enoyl‐CoA hydratase & 3‐hydroxyacyl CoA dehydrogenase	22.7	8.00	1E‐04	1E‐04	−3.23	0.07	0.50	−1.06	0.04	0.99
ACAA1	P09110	Acetyl‐CoA acyltransferase 1	20.6	2.24	3E‐48	2E‐46	−0.24	0.50	0.75	−0.41	0.13	0.99
ACACB	O00763	Acetyl‐CoA carboxylase beta	20.4	2.01	7E‐23	2E‐21	−0.50	0.04	0.50	−0.11	0.80	0.99
SHMT1	P34896	Serine hydroxymethyltransferase 1	19.3	2.47	2E‐54	1E‐52	−0.45	0.05	0.50	0.09	0.78	0.99
AFMID	Q63HM1	Arylformamidase	18.9	1.74	2E‐09	2E‐08	−0.02	0.94	0.98	−0.31	0.55	0.99
SCP2	P22307	Sterol carrier protein 2	18.8	3.39	1E‐103	5E‐101	−0.43	0.11	0.51	−0.28	0.30	0.99
HSD17B4	P51659	Hydroxysteroid 17‐beta dehydrogenase 4	18.7	2.65	2E‐35	8E‐34	−0.30	0.35	0.67	−0.16	0.60	0.99
ALDH2	P05091	Aldehyde dehydrogenase 2 family member	17.8	2.22	2E‐44	1E‐42	−0.44	0.16	0.53	−0.16	0.62	0.99
ME1	P48163	Malic enzyme 1	17.5	2.79	8E‐25	2E‐23	−0.60	0.26	0.61	−0.26	0.43	0.99
ACSS2	Q9NR19	Acyl‐CoA synthetase short chain family member 2	17.5	1.71	1E‐22	2E‐21	−0.36	0.17	0.53	−0.41	0.21	0.99
ACSL1	P33121	Acyl‐CoA synthetase long chain family member 1	16.3	2.20	3E‐71	6E‐69	−0.42	0.07	0.50	−0.36	0.17	0.99
HMGCS1	Q01581	3‐Hydroxy‐3‐methylglutaryl‐CoA synthase 1	15.9	1.93	3E‐16	4E‐15	0.01	0.99	1.00	−0.27	0.43	0.99
AKR1A1	P14550	Aldo‐keto reductase family 1 member A1	15.8	1.92	4E‐33	1E‐31	−0.45	0.03	0.50	−0.06	0.85	0.99
MVK	Q03426	Mevalonate kinase	14.4	1.65	5E‐16	7E‐15	−0.37	0.12	0.51	−0.39	0.35	0.99
AKR1C3	P42330	Aldo‐keto reductase family 1 member C3	13.5	1.64	5E‐25	1E‐23	−0.52	0.08	0.50	−0.25	0.41	0.99
GCLC	P48506	Glutamate‐cysteine ligase catalytic subunit	13.0	2.11	2E‐37	8E‐36	−0.40	0.10	0.51	−0.15	0.60	0.99
ALDH9A1	P49189	Aldehyde dehydrogenase 9 family member A1	12.9	1.81	4E‐36	2E‐34	−0.39	0.11	0.51	−0.13	0.63	0.99
MVD	P53602	Mevalonate diphosphate decarboxylase	12.9	2.26	5E‐25	1E‐23	−0.76	0.06	0.50	−0.20	0.56	0.99
DCXR	Q7Z4W1	Dicarbonyl and L‐xylulose reductase	12.7	2.16	1E‐37	5E‐36	−0.46	0.08	0.50	−0.16	0.59	0.99
ALDH1L1	O75891	Aldehyde dehydrogenase 1 family member L1	12.2	2.82	6E‐45	4E‐43	−0.31	0.34	0.66	−0.17	0.62	0.99
PGM2	Q96G03	Phosphoglucomutase 2	10.7	1.80	1E‐29	5E‐28	−0.29	0.30	0.64	0.05	0.83	0.99
GPT	P24298	Glutamic–pyruvic transaminase	10.6	2.35	8E‐57	8E‐55	−0.45	0.11	0.51	−0.32	0.20	0.99
HIBADH	P31937	3‐Hydroxyisobutyrate dehydrogenase	10.1	2.03	9E‐13	1E‐11	−0.02	0.97	0.99	0.07	0.85	0.99
FDPS	P14324	Farnesyl diphosphate synthase	10.0	2.29	2E‐41	1E‐39	−0.67	0.03	0.50	−0.18	0.52	0.99
IDI1	Q13907	Isopentenyl‐diphosphate delta isomerase 1	10.0	2.10	2E‐24	5E‐23	−0.97	0.01	0.50	−0.43	0.24	0.99
PRPS2	P11908	Phosphoribosyl pyrophosphate synthetase 2	9.6	1.78	2E‐25	6E‐24	−0.50	0.03	0.50	−0.18	0.59	0.99
GFPT1	Q06210	Glutamine–fructose‐6‐phosphate transaminase 1	9.2	1.91	1E‐19	2E‐18	−0.54	0.06	0.50	−0.12	0.75	0.99
CTH	P32929	Cystathionine gamma‐lyase	9.1	1.86	4E‐46	3E‐44	−0.04	0.89	0.95	−0.22	0.35	0.99
HGD	Q93099	Homogentisate 1,2‐dioxygenase	9.1	2.07	2E‐04	9E‐04	−0.27	0.81	0.92	0.40	0.35	0.99
UGP2	Q16851	UDP‐glucose pyrophosphorylase 2	9.0	2.11	2E‐33	8E‐32	−0.74	0.03	0.50	−0.13	0.66	0.99
RPL36	Q9Y3U8	Ribosomal protein L36	8.8	1.65	5E‐07	3E‐06	−0.11	0.84	0.92	−0.14	0.75	0.99
PTS	Q03393	6‐Pyruvoyltetrahydropterin synthase	8.7	3.88	2E‐41	1E‐39	−0.14	0.79	0.91	−0.11	0.73	0.99
ALDH1A1	P00352	Aldehyde dehydrogenase 1 family member A1	8.6	3.44	7E‐58	8E‐56	−0.58	0.23	0.58	−0.48	0.21	0.99
HAL	P42357	Histidine ammonia‐lyase	8.6	2.76	9E‐05	5E‐04	−2.01	0.38	0.68	−1.49	0.09	0.99
FASN	P49327	Fatty acid synthase	8.5	2.70	2E‐47	1E‐45	−0.74	0.01	0.50	−0.18	0.60	0.99
NAMPT	P43490	Nicotinamide phosphoribosyltransferase	8.3	2.50	5E‐47	3E‐45	−0.69	0.03	0.50	0.10	0.68	0.99
HNMT	P50135	Histamine N‐methyltransferase	8.3	1.87	2E‐19	3E‐18	−0.44	0.19	0.56	−0.18	0.66	0.99
GK	P32189	Glycerol kinase	8.2	2.45	4E‐47	3E‐45	−0.58	0.03	0.50	−0.21	0.50	0.99
FABP3	P05413	Fatty acid binding protein 3	7.7	2.11	2E‐79	4E‐77	−0.08	0.68	0.85	−0.23	0.30	0.99
AKR1C1	Q04828	Aldo‐keto reductase family 1 member C1	7.6	2.69	6E‐52	5E‐50	−0.64	0.05	0.50	−0.12	0.73	0.99
TPK1	Q9H3S4	Thiamin pyrophosphokinase 1	7.0	2.10	3E‐14	4E‐13	0.44	0.44	0.72	−0.31	0.37	0.99
SPR	P35270	Sepiapterin reductase	6.8	2.11	2E‐10	2E‐09	−0.50	0.47	0.74	−0.14	0.70	0.99
GPX3	P22352	Glutathione peroxidase 3	6.6	2.46	4E‐40	2E‐38	0.06	0.88	0.95	−0.62	0.02	0.99
SMS	P52788	Spermine synthase	6.5	1.81	2E‐23	6E‐22	−0.31	0.29	0.63	−0.02	0.94	0.99
OLAH	Q9NV23	Oleoyl‐ACP hydrolase	6.0	2.50	5E‐35	2E‐33	−0.67	0.07	0.50	−0.33	0.34	0.99
ENTPD3	O75355	Ectonucleoside triphosphate diphosphohydrolase 3	5.5	2.01	2E‐36	8E‐35	−0.07	0.86	0.94	−0.18	0.53	0.99
ASL	P04424	Argininosuccinate lyase	5.3	1.66	2E‐26	6E‐25	−0.37	0.14	0.52	0.01	0.96	0.99
GALE	Q14376	UDP‐galactose‐4‐epimerase	5.1	2.20	1E‐34	4E‐33	−0.34	0.15	0.52	−0.23	0.50	0.99
GPHN	Q9NQX3	Gephyrin	5.1	1.67	1E‐17	2E‐16	−0.11	0.71	0.87	−0.40	0.26	0.99

### 3.4 Impact of GDM and BMI on Colostrum and Mature Milk Proteome

The differential abundance analysis of the 3496 identified proteins in breast milk, as assessed with an interaction model that included both GDM and BMI effects, revealed that there were no GDM nor any BMI associated differences in individual protein abundances within colostrum or mature milk that remained significant after correction for multiple testing (Tables S4 and S5; Figure S1).

In the subsequent exploratory feature selection utilized to identify if there were proteins that would be best indicative of mothers with or without GDM, we found that the level of one protein, lecithin‐cholesterol acyltransferase (LCAT), was statistically higher in mature milk from mothers with GDM. There were also proteins with higher abundances in mature milk of mothers without GDM, that is, RAS p21 protein activator 1 (RASA1), phospholipase D1 (PLD1), retinoid acid induced 14 (RAI14), and cystathionine beta‐synthase (CBS). Feature selection detected no statistically significant proteins in colostrum associated with GDM.

Thereafter, we examined if there were systematic changes in the protein groups affected by GDM by undertaking a GSEA with both colostrum and mature milk based on our interaction model. In colostrum, we identified 224 GO, KEGG, and Reactome pathways statistically significantly upregulated in the colostrum of mothers with GDM (Table S3). Across the databases, the most commonly upregulated pathway was one linked with the structure of the ribosome.

The level of P21 activated kinase 4 (PAK4, adj. *p* = 0.006) was increased in the colostrum from mothers with higher BMI, while those of complement C1s (C1s, adj. *p* = 0.006) and Complement C1r subcomponent (C1R, adj. *p* = 0.04) were decreased.

We did not find any statistically significant associations between BMI values and proteins in mature milk. GSEA, when assessed in terms of increasing BMI values, revealed 90 upregulated GO, KEGG, and Reactome pathways in colostrum, which shared the dynamics of the actin network facilitated by 19 proteins (Table S3).

In colostrum, the BMI of the mothers with GDM was positively correlated with the abundance of 36 proteins and negatively correlated with the abundance of 13 proteins (Table S6). Among the top positively correlated proteins were S100 calcium‐binding proteins A9 and A8 (S100A9, S100A8). In colostrum, no statistical significance was linked to a change in BMI in mothers without GDM.

In mature milk, the abundance of 25 proteins was increased, whereas those of 14 proteins were decreased in mothers with GDM when their BMI increased (Table S6), proteins calcitonin‐related polypeptide beta (CALCB), calsynthenin 3 (CLSTN3), and major histocompatibility complex, class II, DP alpha 1 (HLA‐DPA1). We found a statistically significant decrease in C‐reactive protein (CRP) and apolipoprotein A1 (APOA1) abundance when BMI increases in mothers with GDM. In the mature milk of mothers without GDM, we found a statistically significant negative correlation in the abundance of clusterin with BMI (Table S6).

GSEA of GDM and increasing BMI identified 26 significant GO, KEGG, and Reactome pathways in colostrum, which share the upregulation of the organization of the actin cytoskeleton (Table S3).

## 4 Discussion

Our study characterized the dynamic change of the breast milk proteome from colostrum to mature milk and demonstrated its adaptability to the effects of GDM and BMI variations on the global proteome, although we identified a few protein abundances being affected by these conditions. Use of optimized analytics [11] allowed us to identify 3496 proteins and their abundance, thus our study presents the most detailed characterization of these type of samples to date. The results may be of significance given the increasing prevalence of GDM, the associated health risks of maternal overweight, and the importance of colostrum and breast milk composition with maturation of the infant immune system, gut development, and future health.

We observed significant changes in the highly expressed proteins from colostrum to mature milk. Namely, immunoglobulins, the polymeric immunoglobulin receptor, and TNC, which play a crucial role in the transmission of passive immunity and support neurodevelopment in infants [31, 32], showed a high abundance in colostrum. It is known that levels of immunoglobulins decrease during lactation [33, 34], which was also observed in our study. In contrast, Lysozyme, FABP3, BSSL, and albumin, which are likely contributing to antimicrobial activity and enzymatic digestion in the infant [35, 36], exhibited a higher abundance in mature milk. Hence, these highly expressed proteins of colostrum may help to protect the immature immune system at the state when a neonate is most vulnerable to infection. After the first months of life, the risk is significantly decreased and the infant is rapidly growing, supported by nutritional mature milk proteins [37].

Another interesting finding in this study is the upregulation of proteins related to adhesion processes in colostrum, which possibly contributing to the integrity of the milk‐derived immune protection in the infant. Adhesion processes are essential for immune cell communication, facilitating interactions between cells of innate immunity and their target sites [38, 39]. This is reflected in our data by the upregulation of clusters of differentiation proteins (CD36, CD47, CD55, and CD74), thrombospondins, SRC, integrins such as ITGA6, and further vascular cell adhesion molecule 1 (VCAM1), PSG2, and CXCL13 were abundant in colostrum. In line with this immunological priming, we identified that proteins involved in the immune system, particularly those associated with antigen processing and presentation via MHC class II and the intestinal immune network for IgA production, were more abundant in colostrum than in mature milk. The MHC II complex transmits information about pathogens to T cells, facilitating their activation and the subsequent triggering of an immune response to promote pathogen elimination, thereby possibly contributing to infant immunity [40, 41]. There are recent studies suggesting that MHC class II complexes play a role in shaping the composition of the gut microbiota, potentially influencing immune regulation and disease susceptibility [42].

In addition to the MHC II complex, mucins are another group of proteins with potential implications for gut function and immune system maturation; these can be viewed as the gatekeepers of the mucosal barrier of the gastrointestinal tract [43]. We detected the upregulation of pathways related to O‐glycan biosynthesis and O‐linked glycosylation of mucins, driven by 12 glycosyltransferases, providing insights into the functions of mucins and their glycosylation processes in colostrum. Several mucins have been identified in our dataset; of these, MUCL1 and MUC1 were among the most abundant proteins. It is well‐established that mucins play a crucial role in host defense by acting as decoys or providing steric hindrance against pathogens, thereby preventing pathogen invasion of the gut [44, 45]. In addition, a study conducted in mice revealed that certain o‐glycosylation patterns of mucins result in the promotion of certain bacterial species in the gut [46]. Thus, maternal mucins may further promote the colonization of the commensal gut microbiota in infants. TLR5 is another protein of interest, since in our data it was abundant in colostrum but poorly detected in mature milk. Previous studies with mice [47, 48, 49] have suggested that TLR5 may be involved in the promotion of colonization by non‐flagellated bacteria. Thus, it may be speculated that a high abundance of TLR5 may play a role in the interactions with gut microbiota in the infant [50].

In mature milk, there were upregulations of several of the proteins involved in fatty acid, cholesterol, and triglyceride synthesis. These findings are consistent with previous studies [51, 52]. These results could be explained by their high prevalence in the mammary gland. In addition, LCAT was positively associated with GDM, while the abundance of apolipoprotein A1 (APOA1) showed an inverse association with increasing BMI in mothers with GDM in mature milk. Similar reductions in colostrum APOA1 levels among mothers with GDM, without adjustment for BMI, have also been reported [12]. APOA1 is the main structural protein of HDL, whereas LCAT catalyzes the synthesis of cholesterol esters within HDL particles; together, these proteins regulate the size of HDL particles [53]. Low plasma APOA1 levels have been associated with an increased risk of cardiovascular disease [54]. Previous research in the same study population revealed that mothers who later developed GDM had elevated serum levels of small HDL particles and reduced levels of large HDL particles [55]. Additionally, elevated LCAT levels in plasma sampled from the umbilical vein from mothers with GDM have been reported, suggesting a potential role in HDL particle remodeling during pregnancy [56]. Thus, these findings suggest that altered HDL metabolism in the breast milk of mothers with GDM and an elevated BMI may reflect their aberrant metabolic profile, with possible implications for both maternal health and neonatal lipid metabolism and a putative long‐term cardiovascular risk. Further research is warranted to confirm this association.

This study demonstrated that GDM and BMI, to some extent, influenced the breast milk proteome. Here, the abundances of two proteins in colostrum, S100A8 and A9, forming the antimicrobial and antifungal calprotectin complex, correlated with the mother's GDM status and her BMI. It has been previously suggested [57] that newborns have elevated fecal calprotectin levels as compared to older children. This elevated concentration usually decreases during the first 9 months of life. Our results indicate that part of this high calprotectin concentration may be induced by external supplementation supplied via the colostrum. Further investigations will be needed to determine whether maternal BMI‐ and GDM‐associated variations in calprotectin levels in milk have functional implications for the maturation of the neonatal gut and immune system. In contrast, we found that the level of the well‐known inflammation marker, CRP, was lower in breast milk from women with GDM and higher BMI values, a finding that is supported by previous reports [13].

### 4.1 Strengths and Limitations

The study's design is a clear strength. This is a prospective study starting from early pregnancy and subsequently onwards that allowed us to collect specific data during pregnancy, including the diagnosis of GDM that could possibly influence the breast milk proteome. Another strength was the application of a novel proteomics approach, which meant that a large number of proteins could be identified. In addition, we enrolled a higher number of participants into our study population in comparison to previous research in the same field. One limitation is the absence of normal weight participants in the study population, which makes it more difficult to identify differences relevant to those mothers with high BMIs. Nonetheless, we utilized a broad spectrum of BMI values. We chose to examine this population of pregnant women with overweight and obesity as they represent a major at‐risk group due to the global prevalence of obesity and its established health risks for both mother and child. One final caveat is that the protein extraction involved a necessary defatting step, that is, we analyzed a defatted supernatant, with mostly soluble proteins, probably leading to a reduced possibility to detect membrane proteins.

### 4.2 Concluding Remarks

These experiments characterized the evolution of the breast milk proteome from colostrum to mature milk. We observed that colostrum was enriched in immune response proteins and mature milk shifting toward the synthesis of lipid‐based proteins to support the infant's changing nutritional needs. Notably, as far as we are aware, our comprehensive proteomic analysis allowed us to undertake the widest breast milk proteome characterization to date. Our novel findings point to the presence of previously unreported associations between maternal GDM and BMI with the specific proteins involved in HDL formation. Furthermore, we provide the first evidence that elevated fecal levels of calprotectin during early infancy may be directly influenced by external supplementation via the colostrum. Given the ever‐increasing prevalence of GDM and obesity, these newly identified proteome alterations have important implications for understanding the development of immunity in the infant as well as its long‐term health outcomes. Further research will be needed to evaluate the impact of these alterations.

## Conflicts of Interest

The authors declare no conflicts of interest.

## Supporting information




**Supporting file 1**: mnfr70225‐sup‐0001‐SuppMat.xlsx

**Supporting file 2**: mnfr70225‐sup‐0002‐SuppMat.xlsx

**Supporting file 3**: mnfr70225‐sup‐0003‐SuppMat.xlsx

**Supporting file 4**: mnfr70225‐sup‐0004‐SuppMat.xlsx

**Supporting file 5**: mnfr70225‐sup‐0005‐SuppMat.xlsx

**Supporting file 6**: mnfr70225‐sup‐0006‐SuppMat.xlsx

**Supporting file 7**: mnfr70225‐sup‐0007‐SuppMat.xlsx

**Supporting file 8**: mnfr70225‐sup‐0008‐SuppMat.docx

## Data Availability

The datasets are not publicly available since they contain information that could compromise participant privacy and consent, but are available from the corresponding author upon reasonable request and subject to a collaboration agreement. The source code is available from: https://zenodo.org/records/16944069.
