# Supplementary material for: Breast Milk Proteome: Changes in the Different Stages of Lactation and Impacts of Gestational Diabetes Mellitus and Body Mass Index
Source: Mol Nutr Food Res. 2025 Sep 1;69(22):e70225. doi: 10.1002/mnfr.70225 (PMC12643191; doi:10.1002/mnfr.70225)
Supplement: Supplementary file 8 — Supporting file 8: mnfr70225‐sup‐0008‐SuppMat.docx [file MNFR-69-e70225-s004.docx]

**Supplemental Material - Evolvement of proteome from colostrum to mature milk and impact of GDM and degree of overweight status**

Timo Seitz*, Jenni Viitaharju*, Chouaib Benchraka, Johannes Merilahti, Marko Kalliomäki, Lauri Polari, Diana Toivola, Leo Lahti, Otto Kauko, Kirsi Laitinen

*T. Seitz and J. Viitaharju

Institute of Biomedicine

Research Centre for Integrative Physiology and Pharmacology

University of Turku

Kiinamyllynkatu 10, Turku FI-20520, Finland

E-mail: ttseit@utu.fi and jessou@utu.fi


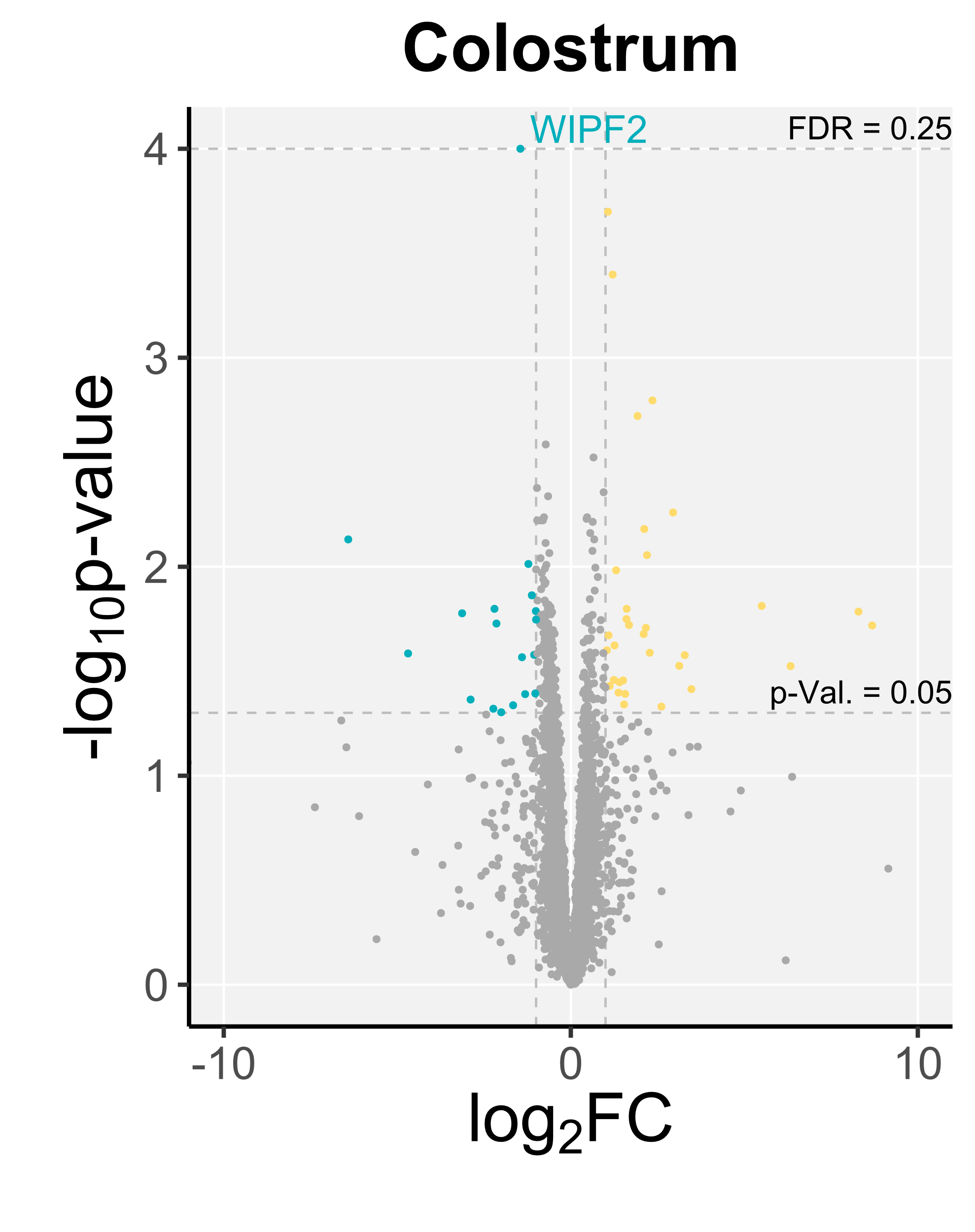

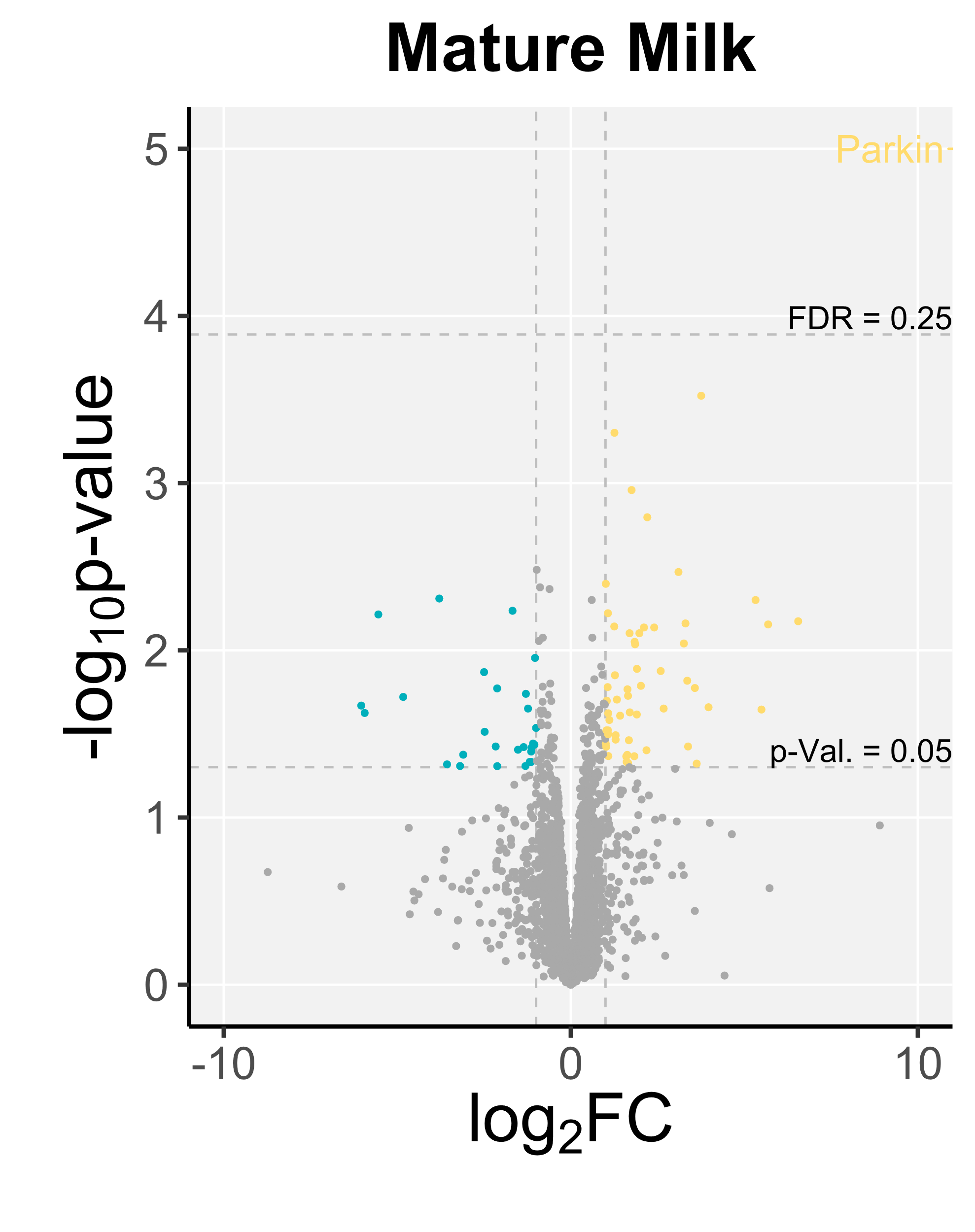


Figure S1. Volcano plots of the results of the differential abundance analysis in the lactational stages with regard to the GDM status using limma R package. Color filter p-value <0.05 and annotation filter FDR <0.25.
